# Psychological Symptoms Among Evacuees From the 2016 Fort McMurray Wildfires: A Population-Based Survey One Year Later

**DOI:** 10.3389/fpubh.2021.655357

**Published:** 2021-05-04

**Authors:** Geneviève Belleville, Marie-Christine Ouellet, Jessica Lebel, Sunita Ghosh, Charles M. Morin, Stéphane Bouchard, Stéphane Guay, Nicolas Bergeron, Tavis Campbell, Frank P. MacMaster

**Affiliations:** ^1^École de Psychologie, Université Laval, Quebec City, QC, Canada; ^2^Alberta Health Services, Calgary, AB, Canada; ^3^Département de Psychoéducation et de Psychologie, Université du Québec en Outaouais, Gatineau, QC, Canada; ^4^Centre de Recherche de l'Institut Universitaire en Santé Mentale de Montréal, Montréal, QC, Canada; ^5^Département de Psychiatrie et d'Addictiologie, Université de Montréal, Montréal, QC, Canada; ^6^Doctors of the World Canada, Montréal, QC, Canada; ^7^Department of Psychology, University of Calgary, Calgary, AB, Canada

**Keywords:** posttraumatic stress disorder, insomnia, substance use disorder, disaster and psychological consequences and risks and interventions and prevention, major depression, generalized anxiety disorder

## Abstract

**Background:** The 2016 wildfires in Fort McMurray (Alberta, Canada) led to a massive displacement of 88,000 people and destroyed 2,400 homes. Although no direct human fatality resulted, many individuals feared for their lives or those of their loved ones.

**Objectives:** (1) To estimate the prevalence of post-traumatic stress, major depressive, insomnia, generalized anxiety, and substance use disorders in the adult population of Fort McMurray 1 year after the evacuation; (2) To identify pre-, peri-, and post-disaster correlates of mental health disorders.

**Methods:** A phone survey using random digit sampling was used to survey evacuees. A total of 1,510 evacuees (response rate = 40.2%, 55.5% women, mean age = 44.11, SD = 12.69) were interviewed between May 9th and July 28th, 2017. Five validated scales were administered: the PTSD Symptoms Checklist (PCL-5), the Insomnia Severity Index (ISI), the depression and anxiety subscales of the Patient Health Questionnaire (PHQ-9, GAD-7), and the CAGE Substance Abuse Screening Tool.

**Results:** One year after the wildfires, 38% had a probable diagnosis of either post-traumatic stress, major depressive, insomnia, generalized anxiety, or substance use disorder, or a combination of these. Insomnia disorder was the most common, with an estimated prevalence of 28.5%. Post-traumatic stress, major depressive and generalized anxiety disorders were almost equally prevalent, with ~15% each. The estimated prevalence of substance use disorder was 7.9%. For all five mental health disorders, having a mental health condition prior to the fires was a significant risk factor, as well as having experienced financial stress or strain due to the economic decline already present in Fort McMurray. Five post-disaster consequences were significant predictors of four of the five disorders: decrease in work, decrease in social life, poorer current health status, increase in drug and alcohol use, and higher level of stress experienced since the fires.

**Conclusion:** One year after the fires, more than one third of the evacuees had clinically significant psychological symptoms, including those of insomnia, post-traumatic stress, depression, anxiety, and substance use. This study helped identify individuals more at risk for mental health issues after a natural disaster and could guide post-disaster psychosocial support strategies.

## Introduction

In a natural disaster, physical injuries and material losses go alongside emotional distress, psychosocial problems as well as mental health disorders which can linger years after the event. The mental health consequences of natural disasters are increasingly recognized, although they remain difficult to assess and to address. Systematic reviews indicate that up to 40% of individuals exposed to a natural disaster will develop stress-related or adjustment disorders such as post-traumatic stress disorder (PTSD), major depression, substance use disorders (SUD), insomnia, or complicated bereavement (1, 2). A general consensus in the disaster-related literature is that mental health issues should be an integral part of the medical and emergency response (2). Monitoring the long-term consequences of disasters on mental health and providing ongoing psychosocial support and specialized mental health care to individuals and communities has been identified as a critical gap by a panel of disaster management experts convened by the World Health Organization in 2018 to identify key emergency and disaster research needs (3).

In North America, wildfires causing damage and evacuations in urban areas have been increasing in the past 30 years, a trend that is thought to continue due to climate change (4). The long-term mental health impacts of wildfires have received relatively limited attention to date. After the 2003 California fires, one study showed that two-thirds of participants had reported having feared for their own life or for the life of a loved one; 3 months after the fires, one quarter of respondents met the criteria for PTSD, and one third met the criteria for major depression (5). One study noted a significant increase in anxiolytic drug consumption after a wave of wildfires in Spain (6). Compared to a control group, persons exposed to wildfires in Greece were observed to have significantly higher somatization, depression and anxiety symptoms, and higher levels of paranoia, hostility, and phobic anxiety (7, 8). Three to 4 years after the Victorian Black Saturday bushfires in Australia (2009), 16–22% of individuals in communities affected by the fires were still found to meet the criteria for PTSD and 13% suffered from major depression (9), indicating that mental health effects are still measurable several years after the event. These studies report precious epidemiological data, but are less informative of the predictors of the development of mental disorders.

In May 2016, a major wildfire affected the Fort McMurray area in Alberta, Canada, destroying 2,400 homes and businesses (10). Approximately 88,000 persons were evacuated. Although there were no deaths directly linked to the wildfire, two persons lost their lives in an accident during the evacuation, and many individuals had to face potential threat to their life or that of their loves ones, for example, by evacuating on roads with fire on both sides, seeing houses on fire or collapsing. A significant number of people were separated from loved ones or experienced important issues with communicating with family members during the evacuation (Thériault, Belleville, Ouellet, and Morin, submitted). Many families were relocated for several months and incurred significant material losses and financial stress.

Three to 5 months after the Fort McMurray fires (July-September 2016), our team collected data on a sample of 379 evacuees with an online questionnaire and conducted standardized psychodiagnostic interviews (in-person or by phone) with a subsample of 55 individuals (11). Although this was a convenience sample, 62.5% were found to have clinically significant symptoms of post-traumatic stress disorder (PTSD) as measured by the PTSD Symptoms Checklist (PCL-5). Among those who completed the psychodiagnostic interview, 29.1% met the criteria for PTSD, 25.5% for major depression, and 43.6% for an insomnia disorder. Another team evaluated 486 residents 6 months after the fires and found that 13% suffered from probable PTSD (15% in females vs. 9% in males) and 20% suffered from probable Generalized Anxiety Disorder (GAD) (12). Taken together, these results indicate rates of mental health issues in the first few months after the disaster that were significantly higher than in the general population.

According to Bonanno (13, 14), reactions to traumatic events are quite heterogeneous and evolve differently across individuals: while many initially experience intense psychological reactions which subside more or less rapidly, other individuals are resilient throughout the aftermath, others still may experience delayed reactions or gradual recovery and some can unfortunately develop chronic mental health issues. Predicting who will develop more serious and pervasive mental health issues requiring clinical attention or psychosocial support is important to increase the preparedness of communities in dealing with the long-term mental health consequences of wildfires and other natural disasters. The level of exposure to the disaster itself and the consequences of the disaster (peri and post-disaster factors) are known to be linked to psychological outcomes. Six months following the Fort McMurray fires, Agyapong et al. (12) found that having witnessed houses being destroyed by the fire, being relocated, and having little support from the family or government were linked to the presence of symptoms of GAD and/or PTSD. Having experienced physical or mental health issues prior to the disaster also increased the risk for mental health issues post-disaster (12, 15). Importantly, the consequences of a natural disaster are often superimposed onto ongoing problems or chronic issues affecting the community (and as such add stress on the individuals), for example, economic uncertainty, psychosocial problems, and difficulties with access to health services, which can interact with post-disaster individual impacts such as job stress, or job relocation (16). In sum, individual and collective psychosocial factors are known to contribute to psychological adaptation after disasters (17).

In a recent review including 40 disasters having occurred between 1982 and 2017, Lowe and colleagues (18) identified several recurring predictors of post-disaster mental health issues: female gender, being at a socioeconomic disadvantage, high exposure during the disaster (e.g., seeing damage or injury first-hand), and having more limited psychosocial resources (18). This review was however limited to PTSD and depression symptoms. Several other mental health outcomes need to be examined including anxiety, insomnia and substance use. Furthermore, it remains unclear which factors are common to the emergence of difference types of psychopathology. It is essential to obtain a clearer picture of the various types of psychological problems that emerge and to identify the predictors of mental health issues, especially potentially modifiable factors which could be intervened upon. Indeed, identifying individuals at greater risk of developing mental health disorders in the long-term after a disaster could help communities better organize psychosocial support or interventions to foster resilience. Such knowledge could be precious in a preventive perspective as wildfires will continue to affect communities in the future. The present study thus aimed to evaluate multiple mental health issues after a disaster and to examine a large spectrum of potential predictors, including pre-, peri, and post-traumatic factors. The specific objectives of this study were (1) to document the presence of probable mental health disorders in the adult population of Fort McMurray 1 year after the evacuation, namely PTSD, major depression, insomnia, GAD and substance misuse and; (2) to identify pre- peri and post-disaster sociodemographic, health and disaster-related factors accounting for symptom severity.

## Method

### Participants and Procedure

To participate in this study, respondents had to be aged 18 or older, be fluent in English, be physically or mentally able to complete the interview and be a current or former Fort McMurray resident who had been evacuated from their home during the 2016 fires. A professional interview firm (BIP Research) drew a random sample of 10,000 home phone numbers and 10,000 mobile phone numbers in Fort McMurray, using the ASDE Sampling Software (ASDE Inc.). Forty-five interviewers worked on the project, supervised by two BIP supervisors as well as the PI (GB), a psychologist specialized in PTSD and a co-PI (MCO), also a certified psychologist. To ensure validity and standardization of the calls, 15% of interview hours were reviewed by a supervisor (315 h). A total of 12,318 numbers were called between May 9th and July 28th, 2017. The response rate was 40.2% and average length of interview was 26.4 min (Table 1). The institutional review board of Université Laval approved the research protocol, and participants provided informed consent.

**Table 1:** Data collection.

**Collection dates: May 9th to July 28th 2017**		
**Interview mean duration: 26 min**		
**Total phone numbers: 12,318**		
	**Frequency**	**Total**
**A. Invalid phone numbers**		**6,237**
Out of service	5,738	
Non-residential	316	
Fax/Modem	183	
**B. Exclusion**		**1,739**
Language	98	
Unable to complete the interview (Age/Disease/Incapacity)	95	
Duplicate (cellular /wired)	115	
Has not been evacuated	1,221	
<18 years old	59	
Out of sector	151	
**C. Undetermined admissibility**		**1,530**
No answer/Voice mail^a^	571	
Refusal before assessment of admissibility	959	
**D. Admissible respondents without complete interviews**		**1,302**
Prolonged absence	0	
Incomplete interview	17	
Appointment after the end of data collection	40	
Refusal after confirmation of admissibility	1,245	
**E. Complete interviews**		**1,510**
	**Response rate^b^^,^^c^**	**$\frac{E}{C*\left(A.R.\;\right)+\;D\;+\;E}=\frac{1,510}{3,757}=0.402$**

a *A phone number is categorized as “no answer” if it has always been unanswered throughout the data collection. For example, an incomplete interview for which there was subsequently no answer at the time of the second appointment is categorized as “incomplete interview” and not as “no answer”*.

b *Computed according to the Canadian Marketing Research and Intelligence Association norms (https://mria-arim.ca/)*.

c *Admissibility Rate (A.R.) $=\frac{D+E}{B+D+E}=\frac{2812}{4551}=\;0.62$*.

### Measures

We developed questions to assess the respondents' socio-demographic characteristics (age, gender, ethnicity, membership in a First Nation, marital status, level of education, work status before the fires, and number of persons depending on the respondent such as children or persons with restricted mobility). Variables describing the status of participants before the fires included: health status (on a 5-point scale ranging from poor to excellent), presence of a serious physical (such as diabetes, heart problems, or cancer), and mental health problems (such as depression, anxiety, or alcohol or drug abuse), and whether the respondent was experiencing financial strain or stress due to the economic turndown in Fort McMurray. Eight questions assessed participants' experience of the fires and evacuation (peri-traumatic variables): we assessed the subjective level of fear experienced during the evacuation (on a 0–10 scale), and whether the respondent was on duty as a first responder. Level of exposure to the traumatic event was assessed by six yes/no questions asking whether the respondent smelled smoke or fire, saw buildings or surroundings on fire, feared for the safety of a loved one, saw explosions or buildings collapsing, feared for their own safety, or was separated from a loved one. Consequences of the fires (or post-traumatic variables) included the subjective level of material loss or damage and the subjective level of stress experienced since the fires (on a 0–10 scale), the number of days evacuated, whether they suffered loss or damages to their household content, their house or apartment, sentimental possessions, car or truck, pets, or other things, changes in work status, whether they returned to live in the same home, activity decrease in work, in sports and leisure, or in social life, current health status (on a 5-point scale ranging from poor to excellent), whether they were having problems with finances and money or with insurance claims, and whether they had increased their alcohol and/or drug use. Descriptive data are presented in Table 2.

**Table 2:** Sample characteristics.

**Sociodemographic characteristics**		
	M	SD
Age	44.11	12.69
	*n*	%
**Gender**		
Female	838	55.50
Male	672	44.50
Other/Prefer not to say	0	0
**Ethnicity**		
White	1,116	73.9
Asian or Pacific Islander	159	10.5
Black or African American	65	4.3
Native American or American Indian	57	3.8
Hispanic or Latino	38	2.5
Metis	27	1.8
Other or Prefer not to say	48	3.2
Member in a First Nation	93	6.2
**Marital Status**		
Married or domestic partnership	1,105	73.2
Single, separated, divorced or widowed	399	26.4
**Level of Education**		
Primary	36	2.4
Secondary	403	26.7
Post-secondary without bachelor degree	597	39.5
Bachelor	342	22.6
Master	113	7.5
Doctoral	15	1.0
**Work Status (before the fires)**		
Full or part time work	1,159	76.8
Homemaker	109	7.2
Retired	87	5.8
Unemployed/out of work/welfare	87	5.7
Student	38	2.5
Sick leave/invalidity	29	1.9
**Number of persons depending on the respondent (e.g., children, persons with restricted mobility)**		
None	611	40.5
1	276	18.3
2	303	20.1
3	174	11.5
4 – 9	139	9.2
**Status before the fires (pre-traumatic)**		
	*n*	%
**Health Status**		
Excellent	447	29.6
Very good	410	27.2
Good	531	35.2
Fair	88	5.8
Poor	34	2.3
Physical health problem	148	9.8
Mental health problem	177	11.7
Financial strain or stress due to economic turndown	346	22.9
**Experience of the fires (peri-traumatic)**		
	M	SD
Subjective level of fear (0–10)	6.51	2.88
	*n*	%
**Level of exposure**		
Smelled smoke or fire	1,456	96.4
Saw buildings or surroundings in fire	1,220	80.8
Feared for the safety of a loved one	1,070	70.9
Saw explosions or buildings collapsing	390	25.8
Feared for their own safety	856	56.7
Separated from a loved one	762	50.5
On duty as first responder	67	4.4
**Consequences of the fires (post-traumatic)**		
	M	SD
Subjective level of material loss or damages (0–10)	2.70	3.28
Subjective level of stress since the fires (0–10)	5.64	3.06
Number of days evacuated^a^	Mean: 46.57 Median: 35 Mode: 30 Range: 0–420	38.89
	*n*	%
**Loss or damages**		
Household content	698	46.2
House or apartment	427	28.3
Sentimental possessions	210	13.9
Car or truck	183	12.1
Pets	66	4.4
Other^b^	188	12.5
Work status change	252	16.7
Returned to live in the same home	1,264	83.7
**Activity decrease**		
Work	461	30.5
Sports and leisure	516	34.2
Social life	596	39.5
**Health status (current)**		
Excellent	336	22.3
Very good	375	24.8
Good	526	34.8
Fair	209	13.8
Poor	64	4.2
Problems with finances or money	427	28.3
Problems with insurance claims	401	26.6
Alcohol/drug use increase	145	9.6

a *51 (3.4%) respondents had not returned home yet at the time of the survey*.

b *Including, in descending order of representativity: Backyard equipment, Lost everything, Recreational vehicles, Rental property, Food, and Clothes*.

We used five validated self-report questionnaires to estimate the prevalence of post-traumatic stress, major depressive, insomnia, generalized anxiety and substance use disorders. The *PTSD Checklist for DSM-5* (PCL-5) (19) is a self-reported questionnaire that assesses post-traumatic symptoms in the last month. It includes 20 items rated on a 5-point Likert scale. Total severity scores range from 0 to 80, with a higher score indicating greater severity. A cutoff score of 33 discriminates between people with or without PTSD and was used to indicate probable PTSD. The *Insomnia Severity Index* (ISI) (20) is a self-reported questionnaire that assesses insomnia symptoms in the last month. It includes seven items scored on a 5-point Likert scale assessing sleep difficulties related to falling asleep, maintaining sleep and early morning awakenings, satisfaction with sleep, interference of problems with daily functioning, the perceptibility of the difficulties associated with sleep problems as well as the level of distress related to the sleep problems. Total severity scores range from 0 to 28, with a higher score indicating greater severity. A cutoff score of 10 is optimal to detect insomnia in a community sample (21) and was used to indicate probable insomnia disorder. The *Patient Health Questionnaire Depression Scale* (PHQ-9) (22) is a self-reported questionnaire that assesses depressive symptoms in the last 2 weeks. It includes nine items on a 4-point Likert scale (from 0 = Not at all to 3 = Nearly every day). Total severity scores range from 0 to 27, with a higher score indicating greater severity. A cutoff score of 10 has been documented as optimal to detect probable Major Depressive Disorder (MDD). The *Patient Health Questionnaire Generalized Anxiety Disorder Scale* (GAD-7) is a 7-item self-reported scale used to identify probable cases of GAD and assess anxiety symptom severity in clinical practice and research (23). It includes seven items rated on a 4-point Likert scale (from 0 = Not at all to 3 = Nearly every day). Total severity scores range from 0 to 21, with a higher score indicating greater severity. A cutoff score of 10 is optimal to screen for GAD in clinical settings (23) and was used to indicate probable GAD. The *CAGE* (Cut down, Annoyed, Guilty, and Eye-opener) (24) is a short 4-item self-reported screening tool to assess substance misuse. Items are answered by no (0) or yes (1). Scores range from 0 to 4, a higher score being an indication of alcohol problems. A total score of two or greater is considered clinically significant.

### Data Analysis

Descriptive statistics were used to describe the study variables. Means and standard deviations (SD) were reported for continuous variables. Frequencies and proportions were reported for categorical variables. The proportions of respondents meeting the cut-off scores as described in the previous section on the various measures were reported to estimate the rates of probable mental disorders in the sample. Generalized linear models (GLM) were used to determine the factors associated with symptom severity (PCL-5, PHQ-9, ISI, GAD-7, and CAGE total scores). Univariate GLM models were first used to determine the factors associated with the outcome variables. Collinearity was checked for the factors, and if the factors were correlated, only one of the factors was used in the model. Standard model building strategies were used to determine the most parsimonious model. Variables significant at *p* < 0.10 level were entered into the multivariate model. The final model for each of the outcome variables was based on statistical significance, with the exception of age, gender, ethnicity, and membership in a First Nation, which were included in all multivariate models regardless of univariate statistical significance. A *p*-value < 0.05 was used to indicate statistical significance. Bonferroni and Holm-Bonferroni (25) corrections were also computed to provide the *p*-values adjusted for multiple testing for each symptom severity. SPSS version 25 (IBM Corp. Released 2017. IBM SPSS Statistics for Windows, Version 25.0. Armonk, NY: IBM Corp.) was used for all statistical analyses.

## Results

### Prevalence of Mental Health Disorders

**Figure 1:**
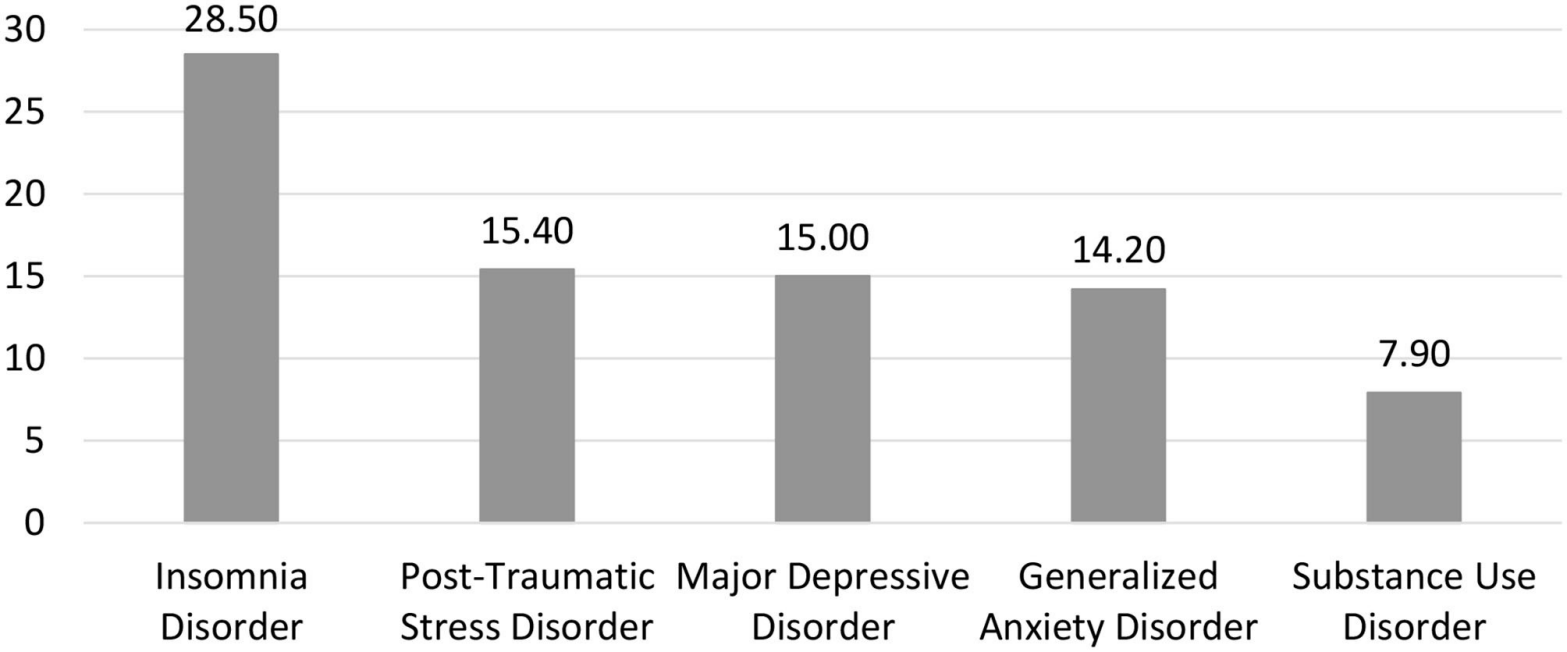
Prevalence estimates of post-traumatic stress, major depressive, insomnia, generalized anxiety, and substance use disorders.

Figure 1 presents the prevalence of probable mental health disorders in the sample. PTSD, MDD and GAD were almost equally represented, with an estimated prevalence of ~15% each. Insomnia disorder was the most common probable diagnosis with a prevalence of 28.5%, while the prevalence of probable SUD was 7.9%.

**Figure 2:**
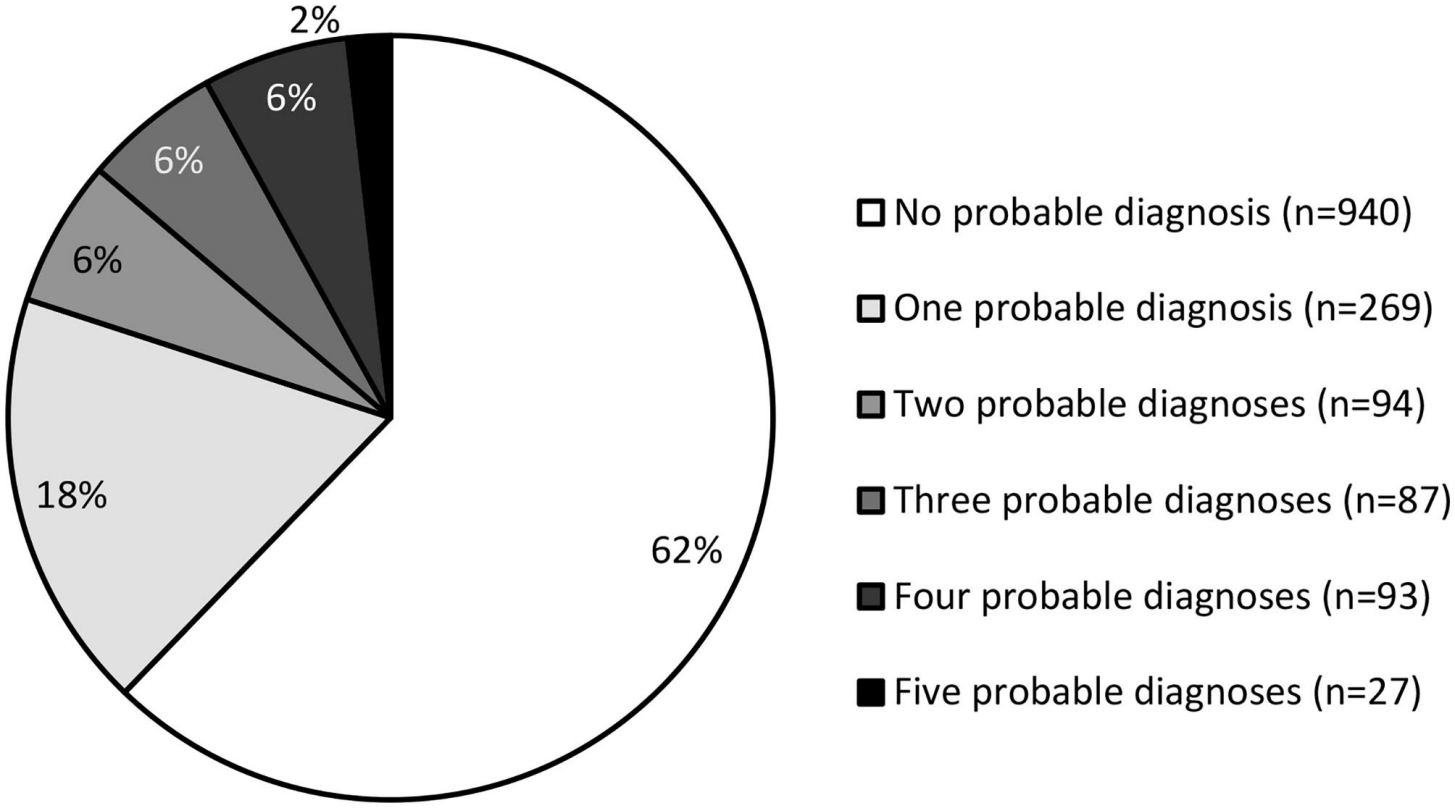
Estimated number of probable diagnoses per person.

More than one third of the sample (37.7%) had at least one probable diagnosis, and 20% had more than one (Figure 2). Among individuals with probable PTSD, 87.1% presented with at least one other probable diagnosis. Among individuals with probable MDD or GAD, this figure increased to 94.3 and 94.0%, respectively. The proportion of individuals with at least one other probable diagnosis was 61.7% among individuals with probable Insomnia Disorder, and 60.0% among individuals with probable SUD.

### Correlates of Mental Health Disorders

Variables associated with univariate or multivariate effects on post-traumatic stress, depressive, insomnia, and anxiety symptom severity, as well as increased risk of drug or alcohol dependency are listed in Tables 3–7, respectively, and summarized in Table 8. For all five mental health disorder symptom severity, having a mental health condition prior to the fires was a significant risk factor, as well as having experienced financial stress or strain due to the economic decline already present in Fort McMurray. After applying Bonferroni and Holm-Bonferroni corrections for multiple testing, the predictive value of prior mental health condition remained statistically significant. Financial stress remained significant only for anxiety after applying the Bonferroni correction. With the Holm-Bonferroni correction, financial stress was predictive of insomnia, anxiety and risk of drug and alcohol dependence. Five post-disaster consequences were significant predictors of four of the five disorder symptom severity: a higher level of stress experienced in the year following the fires, decrease in work, decrease in social life, poorer current health status, and increase in drug and alcohol use. All predictors remained significant after applying corrections for multiple testing, except for decrease in social life, which was no longer a significant predictor of depressive and insomnia symptoms.

**Table 3:** Association between sample characteristics and PTSD symptom severity (PCL-5).

**Characteristic**	**Univariate analysis**		**Multivariate analysis**	
	**B (95% CI)**	***p*-value**	**B (95% CI)**	***p*-value**
Age	0.02 (-0.04 – 0.08)	0.476	0.03 (-0.01 – 0.08)	0.156
Female gender	4.45 (2.89 – 6.01)	<0.0001	0.43 (-0.75 – 1.61)	0.479
Ethnicity: White	3.52 (1.75 – 5.29)	<0.0001	0.27 (-1.04 – 1.58)	0.690
Membership in a First Nation	6.12 (2.89 – 9.35)	<0.0001	1.08 (-1.21 – 3.37)	0.356
Mental health problem	10.43 (8.05 – 12.80)	<0.0001	3.94 (2.21 – 5.66)	<0.0001
Financial strain or stress	8.44 (6.63 – 10.25)	<0.0001	1.54 (0.17 – 2.91)	0.028
Level of fear	2.52 (2.28 – 2.76)	<0.0001	0.69 (0.44 – 0.94)	<0.0001
Feared for the safety of a loved one	11.03 (9.40 – 12.66)	<0.0001	1.73 (0.40 – 3.06)	0.011
Saw explosions or buildings collapsed	8.88 (7.16 – 10.61)	<0.0001	3.34 (2.07 – 4.61)	<0.0001
Level of stress	3.10 (2.90 – 3.31)	<0.0001	1.43 (1.19 – 1.67)	<0.0001
Decrease in work	11.25 (9.65 – 12.85)	<0.0001	2.84 (1.56 – 4.12)	<0.0001
Decrease in social life	14.74 (13.33 – 16.16)	<0.0001	5.47 (4.21 – 6.73)	<0.0001
Health status (compared to Excellent)				
Poor	27.53 (23.95 – 31.10)	<0.0001	12.59 (9.57 – 15.61)	<0.0001
Fair	21.72 (19.41 – 24.03)	<0.0001	12.47 (9.53 – 15.42)	<0.0001
Good	9.37 (7.54 – 11.20)	<0.0001	11.24 (8.43 – 14.05)	<0.0001
Very good	3.89 (1.92 – 5.86)	<0.0001	3.94 (0.96 – 6.91)	0.009
Problems with finances or money	13.81 (12.22 – 15.40)	<0.0001	1.91 (0.47 – 3.34)	0.009
Problems with insurance	8.59 (6.88 – 10.31)	<0.0001	1.04 (-0.24 – 2.31)	0.111

*Variables associated with more severe PCL-5 symptoms in univariate analyses: greater number of persons depending on the respondent, single, divorced or widowed (compared to married or partnership), poorer health status before the fires, saw buildings or surroundings on fire, feared for their own safety, separated from a loved one, greater level of material loss, damage to household content, to house, to sentimental possessions, to cars or trucks, to pets, other damage, work status change, did not return to live in same home, decrease in sports and leisure, increase in alcohol or drug use, greater number of days evacuated*.

*Variables without univariate or multivariate effect: level of education, work status, physical health problem, smelled smoke or fire, on duty as first responder, work status change*.

*Bonferroni p-value = 0.003; Holm-Bonferroni p-value = 0.008*.

**Table 4:** Association between sample characteristics and depression symptom severity (PHQ-9).

**Characteristic**	**Univariate analysis**		**Multivariate analysis**	
	**B (95% CI)**	***p*-value**	**B (95% CI)**	***p*-value**
Age	0.003 (−0.019 – 0.025)	0.791	0.01 (−0.01 – 0.03)	0.184
Female gender	0.91 (0.35 – 1.47)	0.001	0.02 (−0.42 – 0.45)	0.933
Ethnicity: White	0.15 (−0.48 – 0.79)	0.635	0.80 (0.29 – 1.31)	0.002
Membership in a First Nation	1.88 (0.73 – 3.02)	0.001	0.27 (−0.61 – 1.15)	0.543
Single, separated, divorced or widowed	1.59 (0.96 – 2.22)	<0.0001	0.81 (0.32 – 1.30)	0.001
Mental health problem	5.19 (4.36 – 6.01)	<0.0001	2.98 (2.31 – 3.65)	<0.0001
Financial strain or stress	2.92 (2.28 – 3.57)	<0.0001	0.72 (0.19 – 1.25)	0.008
Saw explosions or buildings collapsed	2.35 (1.73 – 2.98)	<0.0001	0.67 (0.19 – 1.16)	0.007
Level of stress	0.88 (0.80 – 0.96)	<0.0001	0.40 (0.32 – 0.49)	<0.0001
Number of days evacuated	0.02 (0.01 – 0.03)	<0.0001	0.006 (0.001 – 0.012)	0.027
Decrease in work	3.64 (3.06 – 4.21)	<0.0001	1.01 (0.51 – 1.51)	<0.0001
Decrease in sports and leisure	4.14 (3.59 – 4.69)	<0.0001	0.87 (0.33 – 1.40)	0.001
Decrease in social life	4.22 (3.69 – 4.75)	<0.0001	0.72 (0.20 – 1.25)	0.007
Health status (compared to Excellent)				
Poor	11.35 (10.09 – 12.60)	<0.0001	6.62 (5.45 – 7.79)	<0.0001
Fair	7.50 (6.68 – 10.09)	<0.0001	3.73 (2.95 – 4.51)	<0.0001
Good	3.36 (2.72 – 4.00)	<0.0001	0.87 (0.27 – 1.47)	0.004
Very good	1.48 (0.78 – 2.17)	<0.0001	0.44 (−0.17 – 1.05)	0.153
Problems with finances or money	4.40 (3.82 – 4.98)	<0.0001	0.77 (0.22 – 1.33)	0.007
Increase in alcohol or drug use	4.74 (3.83 – 5.66)	<0.0001	2.13 (1.40 – 2.87)	<0.0001

*Variables associated with more severe PHQ-9 symptoms in univariate analyses: greater number of persons depending on the respondent, poorer health status before the fires, greater level of fear, saw buildings or surroundings on fire, feared for the safety of a loved one, feared for their own safety, separated from a loved one, greater level of material loss, damage to household content, to house, to sentimental possessions, to cars or trucks, to pets, other damage, work status change, did not return to live in same home, problems with insurance*.

*Variables without univariate or multivariate effect: level of education, work status, physical health problem, smelled smoke or fire, on duty as first responder*.

*Bonferroni p-value = 0.0026; Holm-Bonferroni p-value = 0.006*.

**Table 5:** Association between sample characteristics and Insomnia Symptom Severity (ISI).

**Characteristic**	**Univariate analysis**		**Multivariate analysis**	
	**B (95% CI)**	***p*-value**	**B (95% CI)**	***p*-value**
Age	0.01 (−0.02 – 0.04)	0.401	0.02 (−0.00 – 0.04)	0.098
Female gender	1.22 (0.57 – 1.88)	<0.0001	0.18 (−0.39 – 0.74)	0.538
Ethnicity: White	0.33 (−0.42 – 1.08)	0.388	0.42 (−0.24 – 1.08)	0.210
Membership in a First Nation	1.74 (0.38 – 3.09)	0.012	−0.36 (−1.48 – 0.81)	0.566
Single, separated, divorces of widowed	1.29 (0.55 – 2.04)	0.001	0.70 (0.07 – 1.34)	0.030
Mental health problem	4.54 (3.55 – 5.54)	<0.0001	2.26 (1.38 – 3.13)	<0.0001
Financial strain or stress	3.05 (2.28 – 3.81)	<0.0001	0.98 (0.31 – 1.65)	0.004
Saw explosions or buildings collapsed	2.88 (2.14 – 3.61)	<0.0001	1.06 (0.43 – 1.69)	0.001
Separated from a loved one	2.21 (1.56 – 2.86)	<0.0001	0.86 (0.31 – 1.40)	0.002
Level of stress	1.00 (0.91 – 1.10)	<0.0001	0.49 (0.38 – 0.59)	<0.0001
Number of days evacuated	0.023 (0.014 – 0.031)	<0.0001	0.007 (0.000 – 0.014)	0.059
Decrease in work	3.83 (3.15 – 4.52)	<0.0001	1.16 (0.53 – 1.79)	<0.0001
Decrease in sports and leisure	4.66 (4.01 – 5.31)	<0.0001	1.06 (0.36 – 1.75)	0.003
Decrease in social life	4.65 (4.02 – 5.28)	<0.0001	0.84 (0.16 – 1.52)	0.015
Health status (compared to Excellent)				
Poor	11.51 (9.96 – 13.06)	<0.0001	5.91 (4.39 – 7.42)	<0.0001
Fair	8.12 (7.12 – 9.12)	<0.0001	3.93 (2.92 – 4.94)	<0.0001
Good	4.18 (3.39 – 4.98)	<0.0001	1.38 (0.16 – 2.16)	<0.0001
Very good	2.50 (1.64 – 3.35)	<0.0001	1.23 (0.44 – 2.02)	0.002
Increase in alcohol or drug use	5.01 (3.92 – 6.09)	<0.0001	2.52 (1.56 – 3.47)	<0.0001

*Variable associated with more severe ISI symptoms in univariate analyses: greater number of persons depending on the respondent, poorer health status before the fires, physical health problem, greater level of fear, smelled smoke or fire, saw buildings or surroundings on fire, feared for the safety of a loved one, feared for their own safety, greater level of material loss, damage to household content, to house, to sentimental possessions, to cars or trucks, to pets, other damage, work status change, did not return to live in same home, problems with finances or money, problems with insurance*.

*Variable without univariate or multivariate effect: level of education, work status, on duty as first responder*.

*Bonferroni p-value = 0.0026; Holm-Bonferroni p-value = 0.007*.

**Table 6:** Association between sample characteristics and anxiety symptom severity (GAD-7).

**Characteristic**	**Univariate analysis**		**Multivariate analysis**	
	**B (95% CI)**	***p*-value**	**B (95% CI)**	***p*-value**
Age	−0.01 (−0.03 – 0.01)	0.232	−0.01 (−0.03 – 0.01)	0.176
Female gender	0.86 (0.33 – 1.38)	0.001	−0.47 (−0.90 –−0.04)	0.032
Ethnicity: White	0.39 (−0.21 – 0.98)	0.203	0.62 (0.13 – 1.10)	0.013
Membership in a First Nation	1.77 (0.69 – 2.84)	0.001	0.11 (−0.73 – 0.96)	0.796
Mental health problem	4.09 (3.30 – 4.88)	<0.0001	2.15 (1.51 – 2.80)	<0.0001
Financial strain or stress	2.75 (2.14 – 3.35)	<0.0001	0.81 (0.31 – 1.30)	0.001
Level of fear	0.66 (0.58 – 0.75)	<0.0001	0.16 (0.08 – 0.25)	<0.0001
Level of stress	0.88 (0.81 – 0.95)	<0.0001	0.40 (0.31 – 0.49)	<0.0001
Number of days evacuated	0.020 (0.013 – 0.026)	<0.0001	0.005 (−0.001 – 0.010)	0.075
Damage to house or apartment	2.79 (2.23 – 3.35)	<0.0001	0.76 (0.27 – 1.24)	0.002
Decrease in work	3.38 (2.84 – 3.92)	<0.0001	0.94 (0.47 – 1.40)	<0.0001
Decrease in social life	4.08 (3.59 – 4.58)	<0.0001	1.19 (0.73 – 1.66)	<0.0001
Health status (compared to Excellent)				
Poor	9.29 (8.08 – 10.49)	<0.0001	5.27 (4.15 – 6.38)	<0.0001
Fair	6.97 (6.19 – 7.75)	<0.0001	3.51 (2.76 – 4.25)	<0.0001
Good	3.03 (2.41 – 3.65)	<0.0001	0.62 (0.05 – 1.19)	0.033
Very good	1.25 (0.59 – 1.92)	<0.0001	0.16 (−0.42 – 0.74)	0.588
Problems with insurance	2.73 (2.16 – 3.31)	<0.0001	0.52 (0.04 – 1.00)	0.033
Increase in alcohol or drug use	4.43 (3.58 – 5.29)	<0.0001	1.99 (1.29−2.69)	<0.0001

*Variables associated with more severe GAD-7 symptoms in univariate analyses: greater number of persons depending on the respondent, single, divorced or widowed (compared to married or partnership), poorer health status before the fires, saw buildings or surroundings on fire, feared for the safety of a loved one, saw explosions or buildings collapsed, feared for their own safety, separated from a loved one, greater level of material loss, damage to household content, to sentimental possessions, to cars or trucks, to pets, other damage, work status change, did not return to live in same home, decrease in sports and leisure, problems with finances or money*.

*Variables without univariate or multivariate effect: level of education, work status, physical health problem, smelled smoke or fire, on duty as first responder*.

*Bonferroni p-value = 0.0028; Holm-Bonferroni p-value = 0.006*.

**Table 7:** Association between sample characteristics and increased risk of alcohol or drug dependency (CAGE).

**Characteristic**	**Univariate analysis**		**Multivariate analysis**	
	**B (95% CI)**	***p*-value**	**B (95% CI)**	***p*-value**
Age	−0.006 (−0.011 – 0.002)	0.007	−0.004 (−0.008 – 0.000)	0.028
Female gender	−0.28 (−0.39 – 0.17)	<0.0001	−0.26 (−0.36 – 0.17)	<0.0001
Ethnicity: White	0.07 (−0.08 – 0.22)	0.342	0.11 (−0.02 – 0.24)	0.091
Membership in a First Nation	0.36 (0.13 – 0.58)	0.002	0.18 (−0.2 – 0.38)	0.073
Single, separated, divorces of widowed	0.31 (0.19 – 0.44)	<0.0001	0.13 (0.02 – 0.24)	0.021
Full time or part time work	0.15 (0.01 – 0.29)	0.037	0.14 (0.02 – 0.26)	0.021
Health status before the fires (compared to Excellent)				
Poor	0.76 (0.31 – 1.22)	0.001	0.47 (0.09−0.86)	0.016
Fair	0.24 (−0.01 – 0.49)	0.064	0.18 (−0.04 – 0.40)	0.109
Good	0.08 (−0.07 – 0.22)	0.290	−0.02 (−0.14 – 0.10)	0.777
Very good	0.04 (−0.11 – 0.19)	0.594	−0.02 (−0.14 – 0.11)	0.782
Mental health problem	0.46 (0.29 – 0.63)	<0.0001	0.33 (0.18 – 0.48)	<0.0001
Financial strain or stress	0.24 (0.10 – 0.38)	0.001	0.17 (0.05 – 0.29)	0.005
Increase in alcohol or drug use	1.33 (1.19 – 1.47)	<0.0001	1.26 (1.12 – 1.39)	<0.0001

*Variables associated with higher CAGE score in univariate analyses: lower level of education, greater level of material loss, damage to sentimental possessions, work status change, greater level of stress, decrease in work, in social life, in sports and leisure, poorer current health status, problems with finances or money, problems with insurance*.

*Variables without univariate or multivariate effect: number of persons depending on the respondent, physical health problem, level of fear, smelled smoke or fire, saw buildings or surroundings on fire, feared for the safety of a loved one, saw explosions or buildings collapsed, feared for their own safety, separated from a loved one, on duty as first responder, damage to house or apartment, to household content, to cars or trucks, to pets, other damage, return to live in same home, number of days evacuated*.

*Bonferroni p-value = 0.004; Holm-Bonferroni p-value = 0.006*.

**Table 8:** Summary of regression models.

**Predictors associated with more severe symptoms or higher risk in multivariate models**	**Predicted variables**				
	**PTSD**	**Depression**	**Insomnia**	**Anxiety**	**Drug/Alcohol**
**Sociodemographic characteristics**					
Age					X^*^
Male gender				X^*^	X
Ethnicity: White		X		X^*^	
Membership in a First Nation					
Single, separated, divorced or widowed		X	X^*^		X^*^
Level of education					
Full time or part time work					X^*^
Number of persons depending on the respondent					
**Status before the fires (pre-traumatic)**					
Poorer health status (before the fires)					X^*^
Physical health problem					
Mental health problem	X	X	X	X	X
Financial strain or stress due to economic turndown	X^*^	X^*^	X^†^	X	X^†^
**Experience of the fires (peri-traumatic)**					
Subjective level of fear	X			X	
Smelled smoke or fire					
Saw buildings or surroundings on fire					
Feared for the safety of a loved one	X^*^				
Saw explosions or buildings collapsing	X	X^*^	X		
Feared for their own safety					
Separated from a loved one			X		
On duty as first responder					
**Consequences of the fires (post-traumatic)**					
Subjective level of material loss or damages					
Subjective level of stress since the fires	X	X	X	X	
Number of days evacuated		X^*^			
Loss or damages: household content					
Loss or damages: house or apartment				X	
Loss or damages: sentimental possessions					
Loss or damages: car or truck					
Loss or damages: pets					
Loss or damages: other					
Work status change					
Returned to live in the same home					
Decrease in work	X	X	X	X	
Decrease in sports and leisure		X	X^†^		
Decrease in social life	X	X^*^	X^*^	X	
Poorer health status (current)	X	X	X	X	
Problems with finances or money	X^*^	X^*^			
Problems with insurance claims				X^*^	
Increase in alcohol or drug use		X	X	X	X

*Unadjusted statistical significance p < 0.05*.

* *No longer significant when adjusted for multiple testing (Bonferroni and Holm-Bonferroni)*.

† *Significant when adjusted with Holm-Bonferroni correction, not with Bonferroni correction*.

The variables with predictive power in three of the five models included one sociodemographic characteristic, i.e., being single, separated, or divorced (for models explaining the severity of depressive and insomnia symptoms and the risk of drug or alcohol dependency), and one variable describing the peri-traumatic experience, i.e., having seen explosions or buildings collapse during evacuation (for models explaining the severity of post-traumatic stress, depressive and insomnia symptoms). After applying corrections for multiple testing, marital status remained a significant predictor of depressive symptoms only, and having seen explosions remained a significant predictor of post-traumatic stress and insomnia symptoms.

Socio-demographic characteristics mostly made significant contributions in the multivariate model explaining the risk of drug or alcohol dependency. Interestingly, female gender, a predictor of more severe symptoms in four univariate models, was no longer a significant predictor in multivariate models; on the contrary, female gender had a protective effect in models explaining the severity of anxiety symptoms (no longer apparent after corrections for multiple testing) and the risk of drug or alcohol dependency. Identifying as White was a multivariate predictor of more severe anxiety (no longer apparent after corrections for multiple testing) and depressive symptoms. Membership in a First Nation was associated with more severe symptoms in all five univariate models, but no effect was demonstrated in the multivariate models.

## Discussion

This study aimed to estimate the prevalence of post-traumatic stress, major depressive, insomnia, generalized anxiety and substance use disorders in the adult population of Fort McMurray in a sample of 1,510 evacuees surveyed 1 year after the fires. Insomnia disorder was the most common probable diagnosis, with a prevalence of 28.5%. PTSD, MDD, and GAD were almost equally represented, with an estimated prevalence of ~15% each. The prevalence of probable SUD was 7.9%. All in all, more than one third of the sample (37.7%) had at least one probable diagnosis.

These figures are similar to those reported 6 months after the fires by Agyapong and his colleagues who observed 12.8% PTSD (26) and 19.8% GAD (12) in a smaller and less representative sample of the general adult population in Fort McMurray. The same team evaluated the mental health impacts in several specific Fort McMurray population subgroups 18 months after the fires. In a sample of 3,070 adolescents (grade 7–12), they found 46% of all adolescent students met the criteria for at least one diagnosis 18 months after the disaster (PTSD, depression, GAD or substance misuse (27). In school staff, prevalence rates for probable PTSD were 10.2%, 18.3% for major depression, and 15.7 % for GAD (26). In college students, PTSD affected 11%, major depression 23.4% and GAD 18.7% (28). In primary care patients this time, these researchers rates similar to ours with 13.6% suffering from probable PTSD, and 18% from GAD, but they observed slightly higher prevalence of major depression with 24.8 % in these patients (29).

Our results are also in line with prevalence rates of PTSD (16–22%) and depression (13%) documented 3–4 years after the 2009 bushfires in Australia (9). Taken together, our findings indicate much higher rates of psychopathology among evacuees from forest fires than among the general Canadian population. As a reference, 12-month prevalence rates in Canada for persons 15 and older were of ~4.7% for major depression, 2.6% for GAD, and 4.4% for substance use disorders in 2012 (30). The 1-month prevalence rate for PTSD in Canada was estimated at 2.4% by van Ameringer et al., in 2008 (31). Our study goes further by including insomnia as an important outcome. The vast majority of studies assessing the mental health impacts of fires have focused only on PTSD and depression (32). In a review of 160 studies examining the impacts of disasters reviewed by Norris and collaborators (33), only 10 measured sleep. There is however more and more evidence that persistent sleep problems are among the most frequent reactions after a traumatic event (11, 34–37).

The second objective of the present study was to identify pre-, peri-, and post-traumatic correlates of different mental health disorders. Two pre-existing conditions, that is the presence of a mental health condition and financial problems, were significant predictors of all five types of psychopathology. Not surprisingly, having a mental health condition prior to the fires was a significant predictor in all five models. In non-disaster settings, it is well-known that having a history of mental issues strongly predicts future episodes of psychopathology (38, 39). This seems to be even more true in the context of disasters. As such, particular attention should be given to individuals who are already known to have experienced mental health issues in the past, because their condition can be reactivated or exacerbated. Milligan and McGuinness (40) advocate for early identification of persons suffering from mental illness by first responders in order to ensure that proper referrals are made and appropriate follow-ups are put into place to minimize high-risk behaviors (e.g., suicidal ideation). Furthermore, these authors underscore that individuals with a psychiatric history are in need of specific efforts to stabilize their social environment and ensure they achieve a sense of community. They also suggest that health professionals already involved with these patients can play a pivotal role to help them prepare for the consequences of the disaster. However, one important issue in a disaster such as the one seen in Fort McMurray is that health professionals themselves may have significant needs for psychosocial support as they are part of the affected community (L. Serrano, FuseSocial, personal communication, May 2017).

Having experienced financial stress or strain due to the economic decline already present in Fort McMurray before the fires was another pre-existing condition predicting all five types of psychopathology, although this effect was less resistant to statistical corrections for multiple testing. Several studies have documented that the financial consequences of disasters have an impact on mental health (32): already being in a situation of financial precariousness before facing a disaster also seems important to consider. Before the fires, the Fort McMurray region was already experiencing an economic downturn due to the recession in the Canadian oil sands regions, which was already affecting the use of mental health services (41). The negative impact of economic hardship on mental health (42) and sleep (43) has been documented. The present results go further by suggesting that the financial difficulties present before the fires are crucial in the development of mental health problems, even more so than the direct damage and losses associated with the fires. Future research could investigate whether financial assistance programs contribute reduce the impact of disasters on mental health.

Five post-disaster consequences were significant predictors of four of the five disorders, most often post-traumatic stress, depression, insomnia and anxiety. Decrease in work, decrease in social life, poorer current health status, increase in drug and alcohol use, and the level of stress experienced since the fires were all associated with more severe symptoms, and most of these effects were robust to statistical adjustment for multiple testing. Thus, the results point to the importance of paying particular attention to the level of burden that affects individuals in the year following a natural disaster, whether it be stress, physical health hassles or withdrawal from professional or recreational activities. In fact, in the present study, the level of stress experienced in the year following the fires was found a significant predictor in four psychopathologies vs. two for the level of fear experienced during the event *per se*. This was perhaps because the intensity and the nature of threat to oneself or loved ones could have been considered lower than in other disasters. Indeed, the city of Fort McMurray is surrounded by wilderness, and the city's population is accustomed to the annual presence of forest fires in its vicinities, either wildfires or prescribed burn^1^. In 2016, the wildfires first appeared under control but unexpectedly and rapidly reached the populated areas because of high winds (44). Only an hour after the evacuation was announced, the fire reached the city and blocked one of its two main routes out (45). Another aspect of this event is that although the entire city (~88,000 people) was urgently evacuated, there were no deaths directly related to the fires (although two people died in a car accident while evacuating). In disasters involving more property damage than casualties, post-disaster stress level may be a more important predictor of symptom severity.

The cross-sectional design prevented any conclusion on the causal relationships between significant predictors of the models and the outcomes. It was not possible to determine whether the consequences of fires triggered the symptoms or whether the symptoms led to more severe consequences. For example, did a higher level of perceived stress increase the risk of developing GAD or could it be that the intolerance of uncertainty characterizing people suffering from GAD led to an inflated perception of stress? Another example lies in decreased activity levels and depression which could influence each other bidirectionally and even constitute a vicious cycle. Longitudinal studies with assessments at varying time points post-disaster would be needed to clarify the causal relationships between predictors and mental health outcomes. It would also be important for future studies to distinguish pre-existing conditions from direct psychological consequences of the traumatic event.

It is interesting to note in our sample that female gender, although predicting more severe symptoms in four univariate models, was no longer a significant predictor in the multivariate models. Similarly, First Nations membership was associated with more severe symptoms in all five univariate models, but no effect was demonstrated in the multivariate models. These results contrast with those of other studies that showed that socio-demographic characteristics, particularly female gender, was a predictor of mental health problems such as post-traumatic stress (46), anxiety (47), depression (48), and insomnia (49). Female gender was commonly found a predictor of mental health problems after a disaster (18). When multivariate models are used, however, these sociodemographic characteristics seem to have less predictive power, possibly due to shared variance with other variables.

Our findings suggest the need to provide victims of a natural disaster with effective support and stress management strategies after the more acute phase of the disaster. Multiple clinical practice guidelines, meta-reviews and meta-analyses indicate that psychotherapy, particularly cognitive-behavior therapy (CBT), is an effective and cost-efficient treatment option for PTSD, generalized anxiety, depression, substance misuse, and insomnia. Access to psychotherapy, however is an important preoccupation, especially in the aftermath of a disaster, and there is still limited research indicating how therapy protocols for various mental health issues should be adapted to populations having experienced different types of disasters or other mass traumatic events. Although there are empirically supported treatments for a variety of mental health issues, modalities to increase access to larger numbers of people are still under researched. For example, the potential of online evidence-based interventions could be harnessed, if at all possible to implement, in the longer term after a disaster. This however requires important resources and expertise.

Even if effective treatments are developed for individuals, however, only part of the problem would be solved. Larger-scale social and psychosocial intervention programs are also needed to increase the resilience and preparedness of communities at higher risk of disasters. It is probably reasonable to propose that programs targeting some of the predictors identified in the present study, those which are modifiable at least, could have a positive impact on mental health outcomes: teaching effective stress management techniques, allowing individuals a healthy processing of the fear they experienced during the event, alleviating financial burden, providing opportunities and strategies to return to productive and social activities, providing guidance to healthier consumption of drugs and alcohol, ensuring optimal physical health follow-ups. Furthermore, social and health care systems could identify individuals known to be more vulnerable and who would be in need of closer monitoring in the event of a disaster. Even more important, specific plans could be devised for post-disaster large scale mental health screening and trajectories for accessing mental health support or services. This position echoes that of Rebmann and colleagues who already suggested in 2008 that preventive measures should be put into place to establish plans for accessing mental health *before* a disaster occurs (50). This seems particularly advisable in urban areas where wildfires could be expected to continue to occur or in other communities known to be more at risk for other types of disasters (e.g., flooding). There is still much work to be done in this area however since a systematic review by Roudini et al. in 2017 (51) indicated that very few studies yet have documented community mental health preparedness for disasters or tools that persons or communities could use to prepare themselves for a disaster. These authors suggest that with the increasing numbers of countries facing disasters, local governments and mental health agencies should strive to develop operational plans to intervene, for example by informing different individuals about normal reactions to a disaster and about their potential roles and responsibilities during and after a disaster. The development and evaluation of formal programs which could be implemented at regional or even national levels will need much future work and could focus on educating the public and specific responders or stakeholders on how to best prepare and react in the event of a disaster and in the following years. There is a need to use the growing scientific evidence to support strong advocacy to adopt policies that will build and support community resilience. The present COVID-19 crisis may in fact help us in this regard, since governments are presently rapidly mobilizing themselves to increase access to basic mental health information, self-help tools, crisis helplines, and are identifying resources able to deliver mental health services (52–54).

The results of this study must be interpreted in light of certain limitations. The main one is that sensitive data were collected by interviewers without clinical experience over the phone. Although a portion of the interviews were reviewed by psychologists specializing in the assessment of mental disorders who gave feedback to the interview firm manager, the large number of interviews conducted in a short period of time required a large number of interviewers with a variety of experience level. However, the interview questions and their order were standardized. Also, because the measures were self-reported and targeted potentially sensitive subjects, a social desirability bias cannot be ruled out. Self-reported questionnaires may also overestimate the prevalence of mental health problems when compared to diagnostic interviews, especially in non-clinical populations (55). Last, the cross-sectional design prevented any conclusion on the causal relationships between significant predictors of the models and the outcomes. Despite these limitations, the study built on and extended previous findings by relying on a large randomly selected representative sample. This method made it possible to reach people who had not yet returned to their homes after the disaster (3.4% of the sample), a population that is very difficult to trace in the study of disaster consequences. Finally, the study looked at five mental health outcomes, all of which were assessed using validated instruments.

In conclusion, 1 year after the fires, more than a third of the evacuees showed clinically significant psychological symptoms, including insomnia, post-traumatic stress disorder, depression, anxiety, and substance abuse. This study identified those most at risk for mental health problems after exposure to a natural disaster and could guide the development of psychosocial support strategies after a disaster and increase preparedness for more vulnerable individuals. These results indicate that attention must be paid to the psychiatric past, financial situation and consequences following the traumatic event.

## Data Availability Statement

The raw data supporting the conclusions of this article will be made available by the authors, without undue reservation.

## Ethics Statement

The studies involving human participants were reviewed and approved by Comité D'éthique de la Recherche de l'Université Laval. The patients/participants provided their written informed consent to participate in this study.

## Author Contributions

GB, M-CO, SuG, CM, SB, StG, NB, TC, and FM contributed to the conception and the design of this study. GB, M-CO, JL, and SuG participated in the acquisition of data, analysis, and interpretation of data. GB, M-CO, and JL drafted and revised the article. All authors participated sufficiently in this research to warrant authorship, in agreement with the content of the manuscript, reviewed the article critically, and approved the final version.

## Conflict of Interest

SB is President and shareholder of Cliniques et Développement In Virtuo, a clinic that offers psychotherapy services and distributes virtual reality software. All of this is framed by the conflict of interest management policy of the Université du Québec en Outaouais. CM has received research grants from Idorsia and Canopy Health and served as consultant for Eisai, Merck, Pear Therapeutics, Sunovion, and Weight Watchers. The remaining authors declare that the research was conducted in the absence of any commercial or financial relationships that could be construed as a potential conflict of interest.

## Footnotes

^1^Alberta Wildfire. Available online at: https://wildfire.alberta.ca/.

## References

[1] Neria Y Nandi A Galea S. Post-traumatic stress disorder following disasters: a systematic review. Psychol Med. (2008) 38:467–80. 10.1017/S0033291707001353 17803838 PMC4877688

[2] North CS Pfefferbaum B. Mental health response to community disasters: a systematic review. JAMA. (2013) 310:507–18. 10.1001/jama.2013.107799 23925621

[3] Généreux M Schluter PJ Takahashi S Usami S Mashino S Kayano R. Psychosocial management before, during, and after emergencies and disasters-results from the Kobe Expert Meeting. Int J Environ Res Public Health. (2019) 16:81309. 10.3390/ijerph16081309 31013679 PMC6518049

[4] Schoennagel T Balch JK Brenkert-Smith H Dennison PE Harvey BJ Krawchuk MA. Adapt to more wildfire in western North American forests as climate changes. Proc Natl Acad Sci USA. (2017) 114:4582–90. 10.1073/pnas.1617464114 28416662 PMC5422781

[5] Marshall GN ScheIl TL Elliott MN Rayburn NR Jaycox LH. Psychiatric disorders among adults seeking emergency disaster assistance after a wildland-urban interface fire. Psychiatric Services. (2007) 58:509–14. 10.1176/ps.2007.58.4.509 17412853

[6] Caamano-Isorna F Figueiras A Sastre I Montes-Martínez A Taracido M Piñeiro-Lamas M. Respiratory and mental health effects of wildfires: an ecological study in Galician municipalities (north-west Spain). Environ Health Glob Access Sci Source. (2011) 10:48. 10.1186/1476-069X-10-48 21600035 PMC3224540

[7] Papanikolaou V Adamis D Mellon RC Prodromitis G. Psychological distress following wildfires disaster in a rural part of Greece: a case-control population-based study. Int J Emergency Mental Health. (2011) 13:11–26. 21957753

[8] Papanikolaou V Leon GR Kyriopoulos J Levett J Pallis E. Surveying the ashes: experience from the 2007 Peloponnese wildfires 6 months after the disaster. Prehospital Disaster Med. (2011) 26:79–89. 10.1017/S1049023X11000094 21888727

[9] Bryant RA Waters E Gibbs L Gallagher HC Pattison P Lusher D. Psychological outcomes following the Victorian Black Saturday bushfires. Austr N Zeal J Psychiatry. (2014) 48:634–43. 10.1177/0004867414534476 24852323

[10] Alberta Government. Home Again: Recovery After the Wood Buffalo Wildfire. (2016). Available online at: https://open.alberta.ca/publications/9781460131350 (accessed June 1, 2018).

[11] Belleville G Ouellet MC Morin CM. Post-traumatic stress among evacuees from the 2016 Fort McMurray Wildfires: exploration of psychological and sleep symptoms 3 months after the evacuation. Int J Environ Res Public Health. (2019) 16:91604. 10.3390/ijerph16091604 PMC6540600 31071909

[12] Agyapong VIO Hrabok M Juhas M Omeje J Denga E Nwaka B. Prevalence rates and predictors of generalized anxiety disorder symptoms in residents of fort mcmurray 6 months after a wildfire. Front Psychiatry. (2018) 9:345. 10.3389/fpsyt.2018.00345 30108527 PMC6079280

[13] Bonanno GA. Loss, trauma, and human resilience: have we underestimated the human capacity to thrive after extremely aversive events? Am Psychol. (2004) 59:20–8. 10.1037/0003-066X.59.1.20 14736317

[14] Bonanno GA Mancini AD. Beyond resilience and PTSD: mapping the heterogeneity of responses to potential trauma. Psychol Trauma Theory Res Practice Policy. (2012) 4:74–83. 10.1037/a0017829

[15] Tally S Levack A Sarkin AJ Gilmer T Groessl EJ. The impact of the San Diego wildfires on a general mental health population residing in evacuation areas. Admin Policy Mental Health. (2013) 40:348–54. 10.1007/s10488-012-0425-9 22665076

[16] Kulig J Reimer W Townshend I Edge D Lightfoot N. Understanding Links between Wildfires and Community Resiliency: Lessons Learned for Disaster Preparation and Mitigation. Lethbridge: University of Lethbridge, Faculty of Health Sciences (2011).

[17] Norris FH Sherrieb K Galea S. Prevalence and consequences of disaster-related illness and injury from Hurricane Ike. Rehabil Psychol. (2010) 55:221–30. 10.1037/a0020195 20804265

[18] Lowe SR Bonumwezi JL Valdespino-Hayden Z Galea S. Posttraumatic stress and depression in the aftermath of environmental disasters: a review of quantitative studies published in 2018. Curr Environ Health Rep. (2019) 6:344–60. 10.1007/s40572-019-00245-5 31487033

[19] Weathers FW Litz BT Keane TM Palmieri PA Marx BP Schnurr PP. The PTSD Checklist for DSM-5 (PCL-5): National Center for PTSD. (2013). Available online at: https://www.ptsd.va.gov/professional/assessment/adult-sr/ptsd-checklist.asp (accessed April 15, 2021).

[20] Morin CM. Insomnia: Psychological Assessment and Management. New York, NY: Guilford Press (1993). p. xvii, 238–xvii.

[21] Morin CM Belleville G Bélanger L Ivers H. The Insomnia Severity Index: psychometric indicators to detect insomnia cases and evaluate treatment response. Sleep. (2011) 34:601–8. 10.1093/sleep/34.5.601 21532953 PMC3079939

[22] Kroenke K Spitzer RL Williams JB. The PHQ-9: validity of a brief depression severity measure. J General Internal Med. (2001) 16:606–13. 10.1046/j.1525-1497.2001.016009606.x 11556941 PMC1495268

[23] Spitzer RL Kroenke K Williams JB Löwe B. A brief measure for assessing generalized anxiety disorder: the GAD-7. Archiv Internal Medi. (2006) 166:1092–7. 10.1001/archinte.166.10.1092 16717171

[24] Ewing JA. Detecting alcoholism. The CAGE questionnaire. JAMA. (1984) 252:1905–7. 10.1001/jama.252.14.1905 6471323

[25] Holm S. A simple sequentially rejective multiple test procedure. Scand J Statist. (1979) 6:65–70.

[26] Agyapong VIO Juhas M Omege J Denga E Nwaka B Akinjise I. Prevalence rates and correlates of likely post-traumatic stress disorder in residents of fort mcmurray 6 months after a wildfire. Int J Mental Health Addict. (2019) 1–19. 10.1007/s11469-019-00096-z

[27] Brown MRG Agyapong V Greenshaw AJ Cribben I Brett-MacLean P Drolet J. Significant PTSD and other mental health effects present 18 months after the Fort Mcmurray Wildfire: findings from 3,070 grades 7–12 students. Front Psychiatry. (2019) 10:623. 10.3389/fpsyt.2019.00623 31543839 PMC6728415

[28] Ritchie A Sautner B Omege J Denga E Nwaka B Akinjise I. Long-term mental health effects of a devastating wildfire are amplified by sociodemographic and clinical antecedents in college students. Disast Med Public Health Preparedness. (2020) 87:1–11. 10.1017/dmp.2020.87 32536354

[29] Moosavi S Nwaka B Akinjise I Corbett SE Chue P Greenshaw AJ. Mental health effects in primary care patients 18 months after a major wildfire in Fort McMurray: risk increased by social demographic issues, clinical antecedents, and degree of fire exposure. Front Psychiatry. (2019) 10:683. 10.3389/fpsyt.2019.00683 31620033 PMC6760025

[30] Statistics Canada. Table 1 - Rates of Selected Mental or Substance Use Disorders, Lifetime and 12 Month, Canada, Household Population 15 and Older, 2012. (2012). Available online at: https://www150.statcan.gc.ca/n1/pub/82-624-x/2013001/article/tbl/tbl1-eng.htm (accessed April 15, 2021).

[31] Van Ameringen M Mancini C Patterson B Boyle MH. Post-traumatic stress disorder in Canada. CNS Neurosci Therapeut. (2008) 14:171–81. 10.1111/j.1755-5949.2008.00049.x PMC6494052 18801110

[32] Laugharne J van der Watt G Janca A. After the fire: the mental health consequences of fire disasters. Curr Opin Psychiatry. (2011) 24:72–7. 10.1097/YCO.0b013e32833f5e4e 20844434

[33] Norris FH Friedman MJ Watson PJ Byrne CM Diaz E Kaniasty K. 60,000 disaster victims speak: part I. An empirical review of the empirical literature, 1981–2001. Psychiatry. (2002) 65:207–39. 10.1521/psyc.65.3.207.20173 12405079

[34] van Kamp I van der Velden PG Stellato RK Roorda J van Loon J Kleber RJ. Physical and mental health shortly after a disaster: first results from the Enschede firework disaster study. Eur J Public Health. (2006) 16:253–9. 10.1093/eurpub/cki188 16157614

[35] Babson KA Feldner MT. Temporal relations between sleep problems and both traumatic event exposure and PTSD: a critical review of the empirical literature. J Anxiety Disord. (2010) 24:1–15. 10.1016/j.janxdis.2009.08.002 19716676 PMC2795058

[36] Psarros C Theleritis C Economou M Tzavara C Kioulos KT Mantonakis L. Insomnia and PTSD 1 month after wildfires: evidence for an independent role of the “fear of imminent death.” *Int J Psychiatry Clin Practice*. (2017) 21:137–41. 10.1080/13651501.2016.1276192 28084115

[37] Krakow B Melendrez D Warner TD Dorin R Harper R Hollifield M. To breathe, perchance to sleep: sleep-disordered breathing and chronic insomnia among trauma survivors. Sleep Breathing Schlaf Atmung. (2002) 6:189–202. 10.1055/s-2002-36593 12524572

[38] Karsten J Hartman CA Smit JH Zitman FG Beekman AT Cuijpers P. Psychiatric history and subthreshold symptoms as predictors of the occurrence of depressive or anxiety disorder within 2 years. Br J Psychiatry. (2011) 198:206–12. 10.1192/bjp.bp.110.080572 21357879

[39] Nuggerud-Galeas S Oliván Blázquez B Perez Yus MC Valle-Salazar B Aguilar-Latorre A Magallón Botaya R. Factors associated with depressive episode recurrences in primary care: a retrospective, descriptive study. Front Psychol. (2020) 11:1230. 10.3389/fpsyg.2020.622141 32581978 PMC7290009

[40] Milligan G McGuinness TM. Mental health needs in a post-disaster environment. J Psychosocial Nurs Mental Health Services. (2009) 47:23–30. 10.3928/02793695-20090731-01 19772248

[41] Ritchie A Hrabok M Igwe O Omeje J Ogunsina O Ambrosano L. Impact of oil recession on community mental health service utilization in an oil sands mining region in Canada. Int J Soc Psychiatry. (2018) 64:563–9. 10.1177/0020764018785401 29966476

[42] Selenko E Batinic B. Beyond debt. A moderator analysis of the relationship between perceived financial strain and mental health. Social Sci Med. (2011) 73:1725–32. 10.1016/j.socscimed.2011.09.022 22019305

[43] Hall M Buysse DJ Nofzinger EA Reynolds CF 3rd Thompson W Mazumdar S. Financial strain is a significant correlate of sleep continuity disturbances in late-life. Biol Psychol. (2008) 77:217–22. 10.1016/j.biopsycho.2007.10.012 18055094 PMC2267650

[44] Alary B. Fort McMurray Blaze Among Most ‘Extreme” of Wildfires: Researcher. (2016). Available online at: https://www.folio.ca/fort-mcmurray-blaze-among-most-extreme-of-wildfires-researcher/ (accessed April 15, 2021).

[45] French J. Timeline of Evacuation and Return to Fort McMurray. (2016). Available online at: https://edmontonjournal.com/news/insight/timeline-of-evacuation-and-return-to-fort-mcmurray (accessed April 15, 2021).

[46] Visser E Gosens T Den Oudsten BL De Vries J. The course, prediction, and treatment of acute and posttraumatic stress in trauma patients: a systematic review. J Trauma Acute Care Surg. (2017) 82:1158–83. 10.1097/TA.0000000000001447 28520689

[47] McLean CP Asnaani A Litz BT Hofmann SG. Gender differences in anxiety disorders: prevalence, course of illness, comorbidity and burden of illness. J Psychiatric Res. (2011) 45:1027–35. 10.1016/j.jpsychires.2011.03.006 21439576 PMC3135672

[48] Ferrari AJ Somerville AJ Baxter AJ Norman R Patten SB Vos T. Global variation in the prevalence and incidence of major depressive disorder: a systematic review of the epidemiological literature. Psychol Med. (2013) 43:471–81. 10.1017/S0033291712001511 22831756

[49] Smagula SF Stone KL Fabio A Cauley JA. Risk factors for sleep disturbances in older adults: evidence from prospective studies. Sleep Med Rev. (2016) 25:21–30. 10.1016/j.smrv.2015.01.003 26140867 PMC4506260

[50] Rebmann T Carrico R English JF. Lessons public health professionals learned from past disasters. Public Health Nurs. (2008) 25:344–52. 10.1111/j.1525-1446.2008.00715.x 18666940

[51] Roudini J Khankeh HR Witruk E. Disaster mental health preparedness in the community: a systematic review study. Health Psychol Open. (2017) 4:2055102917711307. 10.1177/2055102917711307 28680695 PMC5489140

[52] Government of Canada. Taking Care of Your Mental and Physical Health During the COVID-19 Pandemic. (2020). Available online at: https://www.canada.ca/en/public-health/services/diseases/2019-novel-coronavirus-infection/mental-health.html (accessed April 15, 2021).

[53] Government of Quebec. Protecting Your Well-Being in the COVID-19 Pandemic. (2020). Available online at: https://www. quebec.ca/en/health/health-issues/a-z/2019-coronavirus/protecting-your- well-being-in-the-covid-19-pandemic/?gclid=CjwKCAiAzNj9BRBDEiwAPs L0d60nKiLNeqq4UwMLq7snChT7TeSyz3eKqYpkcdGBA3Eeru6g6rq3yBoC mCEQAvD_BwE (accessed April 15, 2021).

[54] Centers for Disease Control and Prevention. Coping With Stress. (2020). Available online at: https://www.cdc.gov/coronavirus/2019-ncov/daily-life-coping/managing-stress-anxiety.html (accessed April 15, 2021).

[55] Thombs BD Kwakkenbos L Levis AW Benedetti A. Addressing overestimation of the prevalence of depression based on self-report screening questionnaires. Cmaj. (2018) 190:E44–9. 10.1503/cmaj.170691 29335262 PMC5770251

